# Anxiety Predicts Poor Prognosis in Patients With Hypertrophic Cardiomyopathy

**DOI:** 10.3389/fcvm.2022.890506

**Published:** 2022-05-19

**Authors:** Chao-Jie He, Chang-Lin Zhai, Shao-Dai Huang, Hong-Yan Fan, Ye-Zhou Qian, Chun-Yan Zhu, Hui-Lin Hu

**Affiliations:** ^1^Department of Cardiology, The Affiliated Hospital of Jiaxing University, Jiaxing, China; ^2^Department of Anesthesiology, The Affiliated Hospital of Jiaxing University, Jiaxing, China

**Keywords:** hypertrophic cardiomyopathy, anxiety, sudden cardiac death, heart failure, outcome

## Abstract

**Background:**

Hypertrophic cardiomyopathy (HCM) is the prevalent inherited cardiomyopathy and a major contributor to sudden death and heart failure in young adults. Although depression has been associated with poor prognosis in patients with cardiovascular disease, the relationship between anxiety and HCM clinical outcomes has not been addressed. We aimed to determine the prevalence of anxiety symptoms in patients with HCM and the association between anxiety and adverse prognosis in this population.

**Methods:**

A total of 793 patients with HCM were prospectively enrolled and followed up for a mean of 4.1 years from March 2014 to January 2018. The primary endpoint was sudden cardiac death (SCD) events, and the secondary endpoint was HCM-related heart failure events. Anxiety symptoms were assessed using the Hospital Anxiety and Depression Scale (HADS) during outpatient visits or hospital stays.

**Results:**

Elevated scores on the HADS anxiety subscale (HADS-A ≥ 8) were defined as clinically significant anxiety. SCD and HCM-related heart failure events occurred in 76 and 149 patients, respectively, during the follow-up period. Kaplan–Meier survival curves demonstrated the significant association of anxiety with SCD events (log-rank *P* = 0.012) and HCM-related heart failure events (log-rank *P* = 0.001). Multivariable Cox regression analysis showed anxiety as a predictor of SCD events and HCM-related heart failure events (adjusted hazard ratio [HR] = 1.42, 95% confidence interval [CI] = 1.12–2.04, *P* = 0.03; adjusted HR = 2.9,2 95% CI = 1.73–4.03, *P* < 0.001), independent of conventional risk factors and depression. Besides, patients with comorbid anxiety and depression showed a fourfold higher risk of heart failure events and 3.5-fold higher risk of SCD versus those with neither (adjusted HR = 4.08, 95% CI = 2.76–5.91, *P* < 0.001; adjusted HR = 3.52, 95% CI = 2.24–4.67, *P* < 0.001, respectively).

**Conclusions:**

Anxiety was prevalent among Chinese patients with HCM, and it was independently associated with a higher risk of SCD and HCM-related heart failure events, particularly when comorbid with depression. Psychological assessment and intervention should be considered to alleviate anxiety symptoms in this population.

**Clinical Trial Registration:**

http://www.chictr.org.cn, identifier: ChiCTR2000040759.

## Introduction

Hypertrophic cardiomyopathy (HCM) is the common inherited cardiomyopathy with a prevalence of 1:500 in the general population (1, 2). The majority of HCM cases are asymptomatic, which have an average lifespan, but some cases develop symptoms, such as chest pain, syncope, and palpitations (3). In most cases, a life-long pathological process of progressive and adverse cardiac remodeling is found, characterized by cardiomyocyte disarray, interstitial fibrosis, and wall thinning (4). Previous literature has reported an annual incidence of 1–2% for cardiovascular death in this population, with sudden cardiac death (SCD), end-stage heart failure, and thromboembolism being the major causes of premature death (5, 6). Several clinical features associated with adverse outcomes have been identified, including non-sustained ventricular tachycardia (NSVT), maximum left ventricular wall thickness >30 mm, family history of sudden death, unexplained syncope, left atrial diameter, and left ventricular outflow tract obstruction (LVOTO) (7).

Depression and anxiety are widespread mental disorders that may affect psychological wellbeing and the prognosis of other diseases (8). Anxiety is a strongly negative emotion characterized by feelings of worry and uneasiness, often accompanied by excessive and inappropriate fear and triggered by stimuli perceived as threatening, which differs from depression (9). Anxiety disorder is prevalent worldwide, with an estimated lifetime prevalence of ~29% (10). Accumulating evidence has demonstrated that anxiety might be a potential predictor of prognosis in patients with coronary artery disease (CAD) and heart failure independent of depression (11–13). One study on CAD and anxiety has reported sudden death among men with phobic anxiety, which is six times higher than those without phobic anxiety (14).

A few studies on the psychological health of patients with HCM have revealed that this group is under more psychological distress and impaired quality of life than general population norms (15–17). Previously, our research group has reported that the diagnosis of depression is significantly associated with enhanced risk of SCD and HCM-related heart failure events in patients with HCM (18). However, the impact of anxiety symptoms on the survival of patients with HCM remains unknown. In a secondary analysis of data collected for a published study of depression and adverse outcomes in patients with HCM, we aimed to determine the prevalence of anxiety symptoms and the association between anxiety and prognosis after considering depression in this population.

## Methods

### Study Population and Design

The present study adopted methods described previously (18). In brief, between March 2014 and January 2018, patients who were older than 18 years and fulfilled the diagnostic criteria of HCM were recruited at the Affiliated Hospital of Jiaxing University during outpatient visits or inpatient hospitalizations. The diagnosis of HCM was performed following the current 2014 European Society of Cardiology guidelines and imaging, including echocardiogram, electrocardiograph, cardiac magnetic resonance, family history, and genetic testing if possible (3). Patients were excluded from the study if they had cognitive impairment or had evidence of severe coexisting disease (chronic renal failure, chronic obstructive pulmonary disease, and hepatic dysfunction) or comorbid conditions that had poor survival outcomes and limited follow-up. Ethics approval was granted by the Ethics Committee of the Affiliated Hospital of Jiaxing University in accordance with the Helsinki declaration, and all participants signed written informed consent before enrollment.

### Clinical Characteristics

Sociodemographic, concomitant illnesses, conventional risk factors of SCD, echocardiographic findings, discharge medications, New York Heart Association (NYHA) classification, and laboratory data were extracted from the Haitai electronic patient records system (version 3.0). At study entry, data on variables that are known to be strongly associated with the risk of SCD and are potential confounders, including NSVT, maximum left ventricular wall thickness, family history of sudden death, unexplained syncope, and LVOTO, were collected. Patients with previous cardiac arrest or an estimated 5-year risk of sudden death ≥6% were considered for implantable cardioverter defibrillator (ICD) implantation (19). No particular treatment for anxiety was recommended because no evidence was found for the efficacy and safety of its intervention in the HCM population. Hence, only 17 anxious patients took anxiolytics based on their willingness or previous history of psychological diagnosis.

### Assessment of Anxiety

All participants were interviewed using the Hospital Anxiety and Depression Scale (HADS) for anxious symptoms during their first outpatient clinic or hospitalizations and 12 months later. The HADS is a 14-item self-report questionnaire comprising two 7-item subscales assessing anxiety (HADS-A) and depression (HADS-D) during the past week. Each item is rated from zero to three, yielding a sum score for each subscale of 0–21, with a higher score indicating worse symptoms (20). The instrument was developed to differentiate anxiety from depression at the time of diagnosis in a hospital setting, thereby discarding all vague somatic symptoms and instead comprising the subscale based on the psychological and cognitive symptoms of anxiety. It is a validated and reliable tool in medical practice for screening psychological distress in psychiatric, nonpsychiatric, and well populations. A cutoff score of 8 on the HADS-A subscale (range, 0–21) provides the best compromise between sensitivity and specificity for patients with HCM (17). In previous studies, the HADS-A subscale had excellent internal consistencies (Cronbach's α value of 0.67–0.93) and moderate test–retest reliability (r > 0.8) (21). Norman et al. reported HADS-A with 96% sensitivity and 79% specificity for the HCM population at a cutoff score of 8 (17). In the present study, we defined caseness for anxiety as HADS-A score ≥ 8.

### Primary and Secondary Endpoints

The primary endpoint of interest was SCD events, including sudden death, ICD shock, and documented cardiac arrest. The secondary outcome was HCM-related heart failure events, defined as a composite of heart failure death, cardiac decompensation, heart failure hospitalization, and stroke. The definition of each event was described explicitly in our previous published paper (18). All participants were contacted by telephone, clinical visit, or WeChat semiannually up to 5 years after the initial enrollment to document adverse events and vital status. An endpoint committee consisting of 2 physicians blinded to HADS-A scores reviewed medical records, regulatory documents, and death certificates to adjudicate heart failure and SCD events.

### Statistical Analysis

Continuous data were presented as mean ± SD and analyzed by *t*-test, and categorical data were presented as percentages and analyzed by Chi-square test or Fisher exact test, as appropriate. Univariable and multiple Cox proportional hazards models were applied to examine the association between anxiety and clinical outcomes during an average follow-up of 4.1 years. The multivariable models were performed to adjust for potential confounders using a stepwise selection method (significance level required for entry and stay criteria into the model: *P* < 0.10), forcing the number of established SCD risk factors into a priori model. Thus, variables entered into the multivariable model for SCD events, including unexplained syncope, family history of SCD, NSVT on Holter, LVOTO, maximum LV wall thickness >30 mm, and depression. After the planned model was accomplished, the remaining explanatory variables (i.e., β-blockers and rennin–angiotensin–aldosterone system inhibitors) were retested using sensitivity analysis to verify their influence on effect estimates. Multivariable models were constructed separately for SCD and heart failure events in a similar pattern. Crude and adjusted hazard ratios (HR) with 95% confidence intervals (CI) were estimated from Cox regression analysis. Statistical significance was set at *P* < 0.05. All analyses were performed using SPSS 23.0 Statistics (SPSS Inc., Chicago, IL).

## Results

### Baseline Characteristics

**Figure 1:**
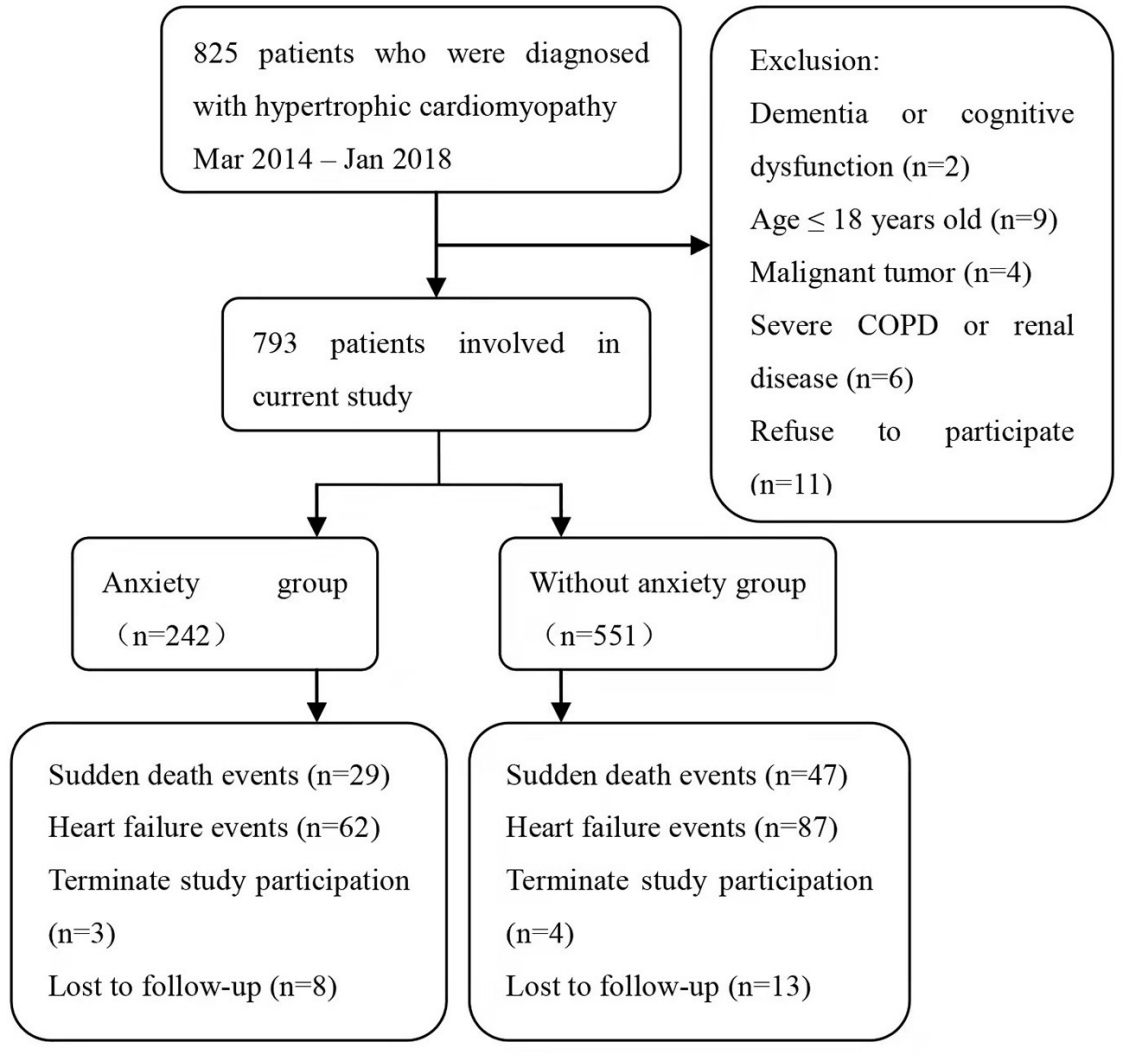
Flowchart of the screening process and dropouts of the present study.

A total of 793 participants who met the diagnostic criteria of HCM were recruited in the present study, of which 242 (30.5%) patients had elevated anxiety symptoms. The average follow-up period was 4.1 years, with a maximum follow-up period of 5 years. Seven hundred and sixty-five participants (96.5%) completed the study, and both groups had similar dropout rates (Figure 1). The mean age was 47.9 years, and 56.2% were men in the overall cohort. Patients with elevated anxiety were more likely to be female and have a history of ICD implantation. The prevalence of other conventional risk factors of SCD was similar between the two groups at baseline. The complete list of clinical and demographic characteristics is shown in Table 1. Notably, seven patients refused further psychiatric assessment after the Structured Clinical Interview for a diagnosis of depression because of exhaustion from assessments or personal reasons.

**Table 1:** Demographic and baseline characteristics of the study population with or without anxiety.

**Characteristics**	**Anxiety (*n* = 242)**	**Without anxiety (*n* = 551)**	***P*** **value**
Demographics			
Age, mean±SD, y	48.2 ± 12.4	47.8 ± 12.6	0.43
Male, *n* (%)	129 (53.3)	317 (57.5)	0.04
BMI, mean±SD, kg/m^2^	24.2 ± 3.6	24.6 ± 3.8	0.29
Maximum LV thickness, mean±SD, mm	20.0 ± 4.6	20.1 ± 4.7	0.67
NYHA class, *n* (%)			
I/II	213 (88.0)	495 (89.8)	0.43
III/IV	29 (12.0)	56 (10.2)	0.43
Comorbid condition, *n* (%)			
Atrial fibrillation	23 (9.5)	51 (9.3)	0.88
Stroke history	4 (1.7)	7 (1.3)	0.21
Diabetes	32 (13.2)	77 (14.0)	0.75
Hypertension	42 (17.4)	99 (18.0)	0.49
Risk factors, *n* (%)			
Non-sustained VT on Holter	46 (19.0)	102 (18.5)	0.70
Unexplained syncope	26 (10.7)	48 (8.7)	0.08
Family history of SCD	32 (13.2)	71 (12.9)	0.66
LVOTO	49 (20.2)	118 (21.4)	0.67
Maximum LV wall thickness ≥ 30 mm	13 (5.4)	27 (4.9)	0.34
Echocardiography, mean ± SD			
LVEF (%)	61.7 ± 6.1	62.8 ± 6.2	0.43
Left atrial diameter	39.9 ± 9.0	40.2 ± 9.0	0.21
ICD implantation			
ICD, n (%)	31 (12.8)	58 (10.5)	0.03
Medications at discharge, *n* (%)			
β-Blockers	148 (61.2)	351 (63.7)	0.38
Calcium channel blockers	42 (17.3)	92 (16.7)	0.59
RAAS inhibitors	39 (16.1)	86 (15.6)	0.72
Diuretic	19 (7.9)	40 (7.3)	0.48
Amiodarone	8 (3.3)	15 (2.7)	0.11
Anxiolytics	17 (7.0)	0 (0)	< 0.001
Laboratory parameters on admission, mean ± SD			
Pro-BNP (pg/mL)	629.2 ± 495.1	602.1 ± 481.7	0.45
Creatinine (umol/L)	74.9 ± 13.7	74.3 ± 13.2	0.59
HADS-A scores	14.1 ± 4.9	5.1 ± 2.7	< 0.001

*SD indicates standard deviation; BMI, body mass index; LV, left ventricle; NYHA, New York Heart Association; VT, ventricular tachycardia; SCD, sudden cardiac death; LVOTO, left ventricular outflow tract obstruction; LVEF; left ventricular ejection fraction; ICD, implantable cardioverter defibrillator; RAAS, rennin-angiotensin-aldosterone system; Pro-BNP, pro-brain natriuretic peptide; HADS-A, Hospital Anxiety and Depression Scale-Anxiety*.

Approximately 30% of the participants reported HADS-A scores at or above the cutoff score of 8 recommended for confirming clinically significant anxiety. HADS-A and HADS-D were highly and positively correlated (*r* = 0.59, *P* < 0.01) in this cohort; 53% of the sample classified as anxiety also met the criteria for depression, and 64% of the depressed patients met the criteria for elevated anxiety. Mean HADS scores in anxious and non-anxious patients were 14.1 ± 4.9 and 5.1 ± 2.7, respectively, at their first assessment and 13.9 ± 4.8 and 5.0 ±2.7, respectively, after 12 months. No statistical differences were observed.

### Clinical Outcomes

In the present study, 76 patients (9.6%) suffered from an SCD event, and 149 (18.8%) experienced an HCM-related heart failure event: 50 sudden deaths, 7 documented cardiac arrests (revived from ventricular tachycardia or ventricular fibrillation), 19 ICD shocks, 19 deaths from heart failure, 21 heart failure hospitalizations, 1 heart transplantation, 13 atrial fibrillation related strokes, and 83 patients with progressive heart failure leading to NYHA functional class III and IV. Of the 29 patients with anxiety and SCD events, 18 died suddenly, 3 survived aborted cardiac arrest, and 8 received appropriate ICD shocks (Table 2).

**Table 2:** Major clinical events of the study participants during follow-up.

**Major clinical events, n (%)**	**Anxiety (*n* = 242)**	**Without anxiety (*n* = 551)**	**HR (95% CI)**
sudden death	18 (7.4)	32 (5.8)	1.6 (1.1–2.7)
Aborted arrest	3 (1.2)	4 (0.7)	1.1 (0.9–1.5)
ICD discharge (VT/VF)	8 (3.3)	11 (2.0)	1.7 (1.2–2.7)
Heart failure death	9 (3.7)	10 (1.8)	2.2 (1.7–3.8)
Heart transplantation	0 (0)	1 (0.2)	1.6 (0.2–12.4)
HCM-related stroke	6 (2.5)	7 (1.3)	1.9 (1.3–3.3)
Progression to NYHA class III/IV	34 (14.0)	49 (8.9)	1.8 (1.4–3.7)
Noncardiac death	4 (1.7)	8 (1.5)	1.0 (0.8–1.2)
Heart failure hospitalization	9 (3.7)	12 (2.2)	1.5 (1.2–3.0)

*HR indicates hazard ratio; CI, confidence interval; HCM, hypertrophic cardiomyopathy; ICD, implantable cardioverter defibrillator; VT, ventricular tachycardia; VF, ventricular tachycardia; NYHA, New York Heart Association*.

### Association Between Anxiety and Clinical Outcomes

**Figure 2:**
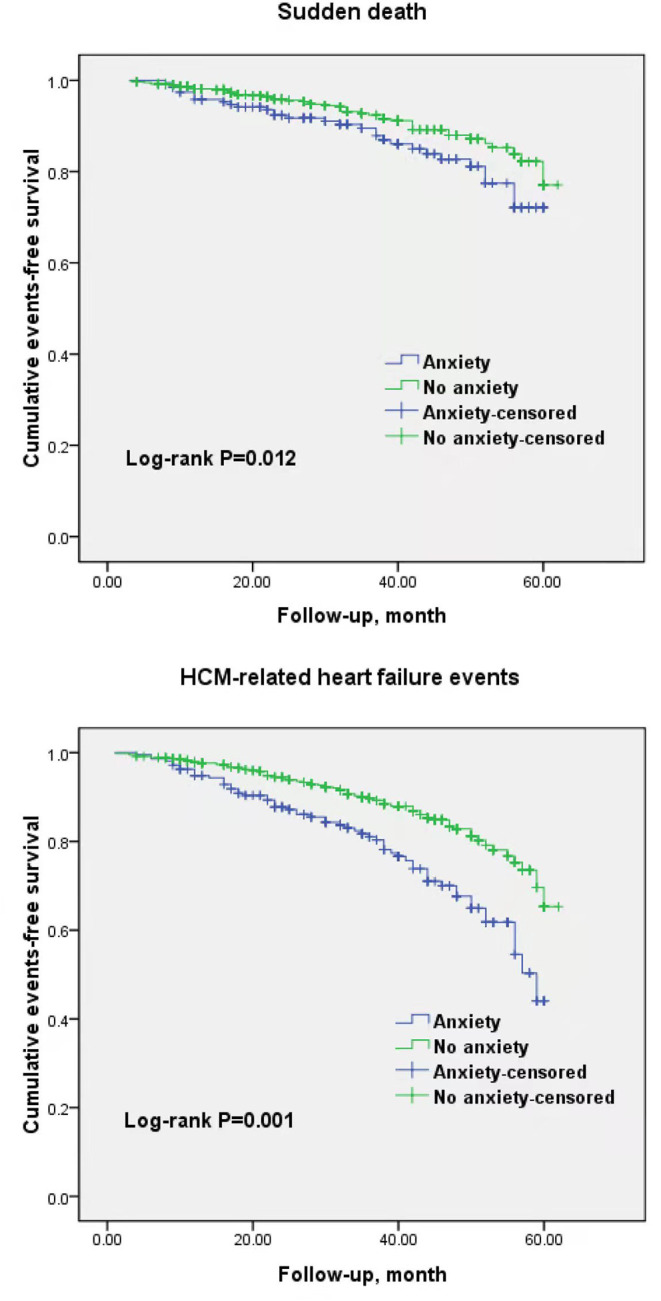
Kaplan–Meier curves for sudden cardiac death events and hypertrophic cardiomyopathy related heart failure events in participants with and without anxiety.

Kaplan–Meier survival analysis showed a greater cumulative SCD (log-rank *P* = 0.012) and HCM-related heart failure (log-rank *P* = 0.001; Figure 2) event-free survival for patients who had low anxiety scores. In univariable Cox regression analysis, anxiety was associated with a significant rate of SCD and heart failure events (unadjusted HR = 1.62, 95% CI = 1.19–2.23, *P* < 0.01; unadjusted HR = 3.08, 95% CI = 1.78–4.23, *P* < 0.001, Table 3). After adjustment for medications, conventional risk factors, and depression in multivariable analysis, anxiety remained an independent prognostic factor for SCD events (adjusted HR = 1.42, 95% CI = 1.12–2.04, *P* = 0.03). Meanwhile, the association between anxiety and heart failure events was maintained (adjusted HR = 2.92, 95% CI = 1.73–4.03, *P* < 0.001), but depression had an attenuated association with increased risk of heart failure events (adjusted HR = 1.27, 95% CI = 1.14–1.94, *P* = 0.04, Table 4).

**Table 3:** Univariable Cox regression of variables influencing SCD events and HCM-related heart failure events.

	**SCD events**		**HCM-related heart failure events**	
**Variables**	**HR (95% CI)**	***P*** **value**	**HR (95% CI)**	***P*** **value**
Age (per decade increase)	0.95 (0.87–1.04)	0.43	1.07 (0.98–1.21)	0.12
Left atrial diameter	1.10 (0.91–1.19)	0.31	1.22 (0.81–1.34)	0.27
Atrial fibrillation	1.11 (0.91–1.14)	0.29	1.24 (1.09–1.54)	0.11
β-Blockers	0.91 (0.72–1.04)	0.05	0.78 (0.69–0.90)	0.02
Calcium channel blockers	0.92 (0.84–1.06)	0.21	0.96 (0.83–1.14)	0.65
RAAS inhibitors	0.95 (0.92–1.02)	0.26	0.88 (0.72–1.02)	0.08
Diuretic	1.01 (0.92–1.05)	0.83	0.95 (0.82–1.09)	0.31
NVT on ambulatory Holter	2.66 (1.95–3.51)	<0.001	1.62 (1.10–2.29)	<0.01
Unexplained syncope	1.77 (1.27–2.82)	0.02	1.12 (0.81–1.18)	0.39
Family history of SCD	1.73 (1.34–2.24)	0.03	1.07 (0.95–1.17)	0.63
LVOTO	1.92 (1.24–2.70)	<0.001	2.50 (1.82–4.19)	<0.001
MLVWT ≥ 30 mm	1.48 (1.23–2.19)	0.02	1.52 (1.04–2.43)	0.03
Depression	2.14 (1.83–2.47)	<0.001	1.78 (1.37–2.25)	<0.001
Anxiety	1.62 (1.19–2.23)	<0.01	3.08 (1.78–4.23)	<0.001

*SCD indicates sudden cardiac death; HCM, hypertrophic cardiomyopathy; HR, hazard ratio; CI, confidence interval; RAAS, rennin-angiotensin-aldosterone system; NVT, Non-sustained ventricular tachycardia; LVOTO, left ventricular outflow tract obstruction; MLVWT, maximum left ventricle wall thickness*.

**Table 4:** Multivariable Cox regression of variables influencing SCD events and HCM-related heart failure events.

	**SCD events**		**HCM-related heart failure events**	
**Variables**	**HR (95% CI)**	***P*** **value**	**HR (95% CI)**	***P*** **value**
β-Blockers	0.82 (0.61–1.01)	0.05	0.82 (0.63–0.92)	0.01
RAAS inhibitors	NA	NA	0.92 (0.85–1.23)	0.13
NVT on ambulatory Holter	2.53 (1.91–3.25)	<0.001	1.37 (1.08–2.09)	0.02
Unexplained syncope	1.62(1.20–2.17)	0.04	1.22 (0.91–1.77)	0.35
Family history of SCD	1.82 (1.35–2.51)	<0.001	1.04 (0.91–1.27)	0.77
LVOTO	2.01(1.60–2.93)	<0.001	2.39 (1.90–3.02)	<0.001
MLVWT≥ 30 mm	1.52 (1.27–1.88)	0.02	1.53 (1.18–2.21)	0.03
Depression	2.33 (1.64–2.93)	<0.001	1.27 (1.14–1.94)	0.04
Anxiety	1.42 (1.12–2.04)	0.03	2.92 (1.73–4.03)	<0.001

*SCD indicates sudden cardiac death; HCM, hypertrophic cardiomyopathy; HR, hazard ratio; CI, confidence interval; RAAS, rennin-angiotensin-aldosterone system; NA, not applicable; NVT, Non-sustained ventricular tachycardia; LVOTO, left ventricular outflow tract obstruction; MLVWT, maximum left ventricle wall thickness*.

We also constructed a model to evaluate how anxiety and depression work together to predict poor clinical outcomes. Consequently, patients with comorbid anxiety and depression showed a fourfold higher risk of heart failure events and 3.5-fold higher risk of SCD versus those with neither (HR = 4.08, 95% CI = 2.76–5.91, *P* < 0.001; HR = 3.52, 95% CI = 2.24–4.67, *P* < 0.001, respectively, Table 5).

**Table 5:** Multivariable analyses for the association between anxiety and depression and the risk of SCD events and HCM-related heart failure events.

**Mental health condition, n**	**SCD events, *n* (%)**	**Adjusted HR (95% CI)**	***P*** **value**	**Heart failure events, *n* (%)**	**Adjusted HR (95% CI)**	***P*** **value**
No anxiety, no depression (*n* = 479)	22 (4.6%)	Reference	NA	42 (8.8%)	Reference	NA
Anxiety only (*n* = 114)	14 (12.3%)	1.77 (1.33–2.83)	0.015	36 (31.6%)	2.89 (1.79–3.93)	<0.001
Depression only (*n* = 72)	13 (18.1%)	2.69 (1.56–3.67)	<0.001	21 (29.2%)	2.72 (1.57–3.78)	<0.001
Both anxiety and depression (*n* = 128)	27 (21.1%)	3.52 (2.24–4.67)	<0.001	50 (39.1%)	4.08 (2.76–5.91)	<0.001

*SCD indicates sudden cardiac death; HCM, hypertrophic cardiomyopathy; HR, hazard ratio; CI, confidence interval; NA, not applicable*.

## Discussion

In the present cohort of the HCM population, approximately one-third of participants experienced anxiety. Those with elevated anxiety symptoms indicated a 1.5-fold increased risk of SCD events and threefold increased risk of HCM-related heart failure events, indicating that anxiety may be a clinical predictor independent of known risk factors and depression disorders. The increased SCD risk was most pronounced in the group with comorbid anxiety and depression, in whom the risk of SCD events was 3.52-fold greater than those with neither. In our earlier work concerning the association between depression and adverse outcomes in patients with HCM, these populations had approximately two times the risk of SCD and heart failure events during the follow-up period (18). Although anxiety has not been identified as a strong predictor of SCD events, the risk of heart failure events was up to three times than depression.

In a recent meta-analysis of 44 studies, anxiety was associated with a moderately increased risk of mortality and poor cardiac outcomes in patients with stable CAD, although this relationship may be explained partly by other clinical covariates (22). However, studies have yielded inconsistent results in patients with heart failure. In two prospective studies conducted over a decade ago, anxiety was marginally associated with mortality in unadjusted analyses, whereas this relationship became non-significant when controlling for relevant factors (12, 13). Considerable literature has focused on the association between anxiety disorders and cardiovascular health. Most studies have reported that generalized anxiety disorder (GAD) is associated with poor outcomes in all stages of CAD, whereas panic disorder (PD) carries a significantly increased risk of the development and progression of cardiac disease (23–25).

However, none of these studies have investigated the impact of anxiety on HCM outcomes or evaluated whether anxiety and depression interact. Our findings indicate the high prevalence of anxiety in the HCM population, which is generally consistent with previous studies. More than two decades ago, Cox et al. first reported a high prevalence of anxiety problems (up to 47.9%) in patients with HCM and concluded that this population was associated with substantial restrictions in health-related quality of life and psychological well-being (15). Ten years later, high prevalence rates of 39% and 37% were reported by Norman et al. and Morgan et al., respectively (16, 17). Notably, the assessment questionnaires of all the abovementioned studies were HADS.

Previous literature has reported that patients with comorbid anxiety and depression had a greater functional impairment and more resistance to psychotherapy than individuals with either condition alone (26). Our findings regarding additive effects between anxiety and depression and SCD and heart failure risk are consistent with previous studies. That is, our findings highlight the prognostic significance of mental disorders and the importance of screening for psychological symptoms in patients with HCM before ICD recommendation. In an investigation of the effects of alcohol septal ablation on psychological distress, depression and anxiety were significantly improved in patients with HCM (27). In addition, previous studies have shown that those attending cardiac genetic clinics were better adjusted, and they worry less. A small group of patients with mood disorders might benefit from education, counseling, and support in specific HCM clinics (28, 29). These findings emphasized the importance of psychological wellbeing and quality of life in these populations.

The mechanism underlying the association of anxiety and adverse clinical outcomes remains unclear. Anxious patients appear less likely to engage in health behavior, indicating the association between anxiety symptoms and cardiovascular health. Mason et al. (30) reported that patients with GAD or PD have higher odds of obesity, diabetes, and substance abuse (31). In addition, chronic stress can lead to increased sympathetic outflow, disrupted heart rate variability and baroreflex sensitivity, and interrupted autonomic nervous system by reducing the vagal tone, which have been linked to increased adverse cardiovascular outcomes of ventricular arrhythmia and SCD in patients with post-acute myocardial infarction and heart failure (32–34). Other potential mechanisms include the association of anxiety with chronically elevated catecholamine levels and inflammatory markers, increased platelet aggregation, and endothelial dysfunction (35). Finally, antidepressant medications including selective serotonin reuptake inhibitors have been associated with a modest effect on the QT interval, with citalopram appearing to cause the greatest QTc prolongation (36, 37).

In the present study, we hypothesized that the first diagnosis of HCM might increase psychological stress at recruitment and assessed anxious symptoms at baseline and after 12 months. No statistical difference was found in HADS-A scores. In addition, the conventional risk factors that had a significant impact on poor prognosis, such as unexplained syncope, family history of SCD, NSVT on Holter, LVOTO, maximum LV wall thickness >30 mm, and depression disorders, were all adjusted for analysis (3, 18). Finally, an instant messaging application, namely, WeChat, improved the accessibility and convenience of follow-up and reduced the dropout rate, verifying the reliability of our conclusions.

Several limitations hinder the generalization of the current findings to all HCM patients. First, almost half of the patients had a first diagnosis of HCM in the present study, including individuals with elevated anxiety. In addition, among patients who have undergone implantation of an ICD to prevent lethal arrhythmias, elevated anxiety levels are present in ~20–40% (38). Second, the assessment of anxiety during hospitalization or clinic visit may influence some individuals, although the HADS was explicitly designed to identify cases of anxiety and depression in hospital settings. Third, we did not collect confounding factors such as substance abuse, socioeconomic status, stress, or physical exercise. Fourth, patients who refused to participate in the study may be more anxious and depressive. Fifth, the HADS is a validated tool, and a cutoff score of 8 was not used to diagnose psychological disorders but to identify high-risk anxiety groups. Sixth, patients with elevated anxiety were more likely to be female, which suggests a selection bias.

## Conclusions

Anxiety is a common mental disorder with a high prevalence in Chinese patients with HCM. In addition, anxiety is strongly associated with an increased risk of SCD and heart failure events, particularly when comorbid with depression. Further study should be considered to validate whether psychological assessment and anxiety intervention can alleviate adverse prognosis in this population.

## Data Availability Statement

The raw data supporting the conclusions of this article will be made available by the authors, without undue reservation.

## Ethics Statement

The studies involving human participants were reviewed and approved by the Ethics Committee of the Affiliated Hospital of Jiaxing University. The patients/participants provided their written informed consent to participate in this study.

## Author Contributions

C-JH: conceptualization, methodology, investigation, and writing-original draft. C-LZ: data curation, formal analysis, writing-original draft preparation, project administration, and writing- reviewing and editing. S-DH: resources, visualization, and investigation. H-YF: formal analysis, validation, and resources. Y-ZQ: software and validation. C-YZ: conceptualization, methodology, and supervision. All authors contributed to the article and approved the submitted version.

## Funding

This research was funded by Project of the Affiliated Hospital of Jiaxing University (2021YJKY008), Provincial-Municipal Joint Construction of Key Medical Disciplines in Zhejiang Province (2019-ss-xxgbx), Zhejiang Medical Association Clinical Research Fund Grant No. 2020ZYC-A45, Pioneer innovation team of Jiaxing Arteriosclerotic Diseases Research Institute (XFCX–DMYH), Jiaxing Institute of Arteriosclerotic Diseases (2020-dmzdsys), and Peak Discipline Established by the First Hospital of Jiaxing (2021-GFXK-02).

## Conflict of Interest

The authors declare that the research was conducted in the absence of any commercial or financial relationships that could be construed as a potential conflict of interest.

## Publisher's Note

All claims expressed in this article are solely those of the authors and do not necessarily represent those of their affiliated organizations, or those of the publisher, the editors and the reviewers. Any product that may be evaluated in this article, or claim that may be made by its manufacturer, is not guaranteed or endorsed by the publisher.
